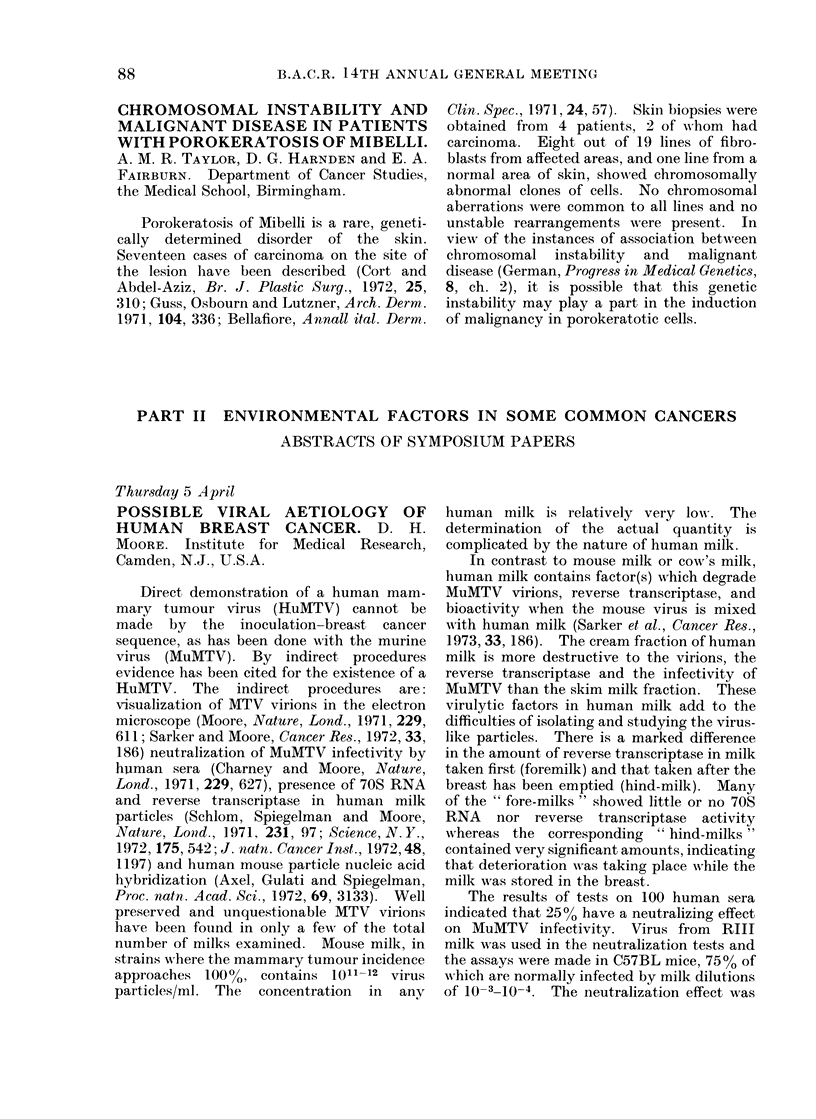# Chromosomal instability and malignant disease in patients with Porokeratosis of Mibelli.

**DOI:** 10.1038/bjc.1973.114

**Published:** 1973-07

**Authors:** A. M. Taylor, D. G. Harnden, E. A. Fairburn


**CHROMOSOMAL INSTABILITY AND MALIGNANT DISEASE IN PATIENTS WITH POROKERATOSIS OF MIBELLI.** A. M. R. TAYLOR, D. G. HARNDEN and E. A. FAIRBURN. Department of Cancer Studies, the Medical School, Birmingham.

Porokeratosis of Mibelli is a rare, genetically determined disorder of the skin. Seventeen cases of carcinoma on the site of the lesion have been described (Cort and Abdel-Aziz, *Br. J. Plastic Surg.*, 1972, **25**, 310; Guss, Osbourn and Lutzner, *Arch. Derm.* 1971, **104**, 336; Bellafiore, *Annall ital. Derm. Clin. Spec.*, 1971, **24**, 57). Skin biopsies were obtained from 4 patients, 2 of whom had carcinoma. Eight out of 19 lines of fibroblasts from affected areas, and one line from a normal area of skin, showed chromosomally abnormal clones of cells. No chromosomal aberrations were common to all lines and no unstable rearrangements were present. In view of the instances of association between chromosomal instability and malignant disease (German, *Progress in Medical Genetics*, **8**, ch. 2), it is possible that this genetic instability may play a part in the induction of malignancy in porokeratotic cells.